# Supplementary material for: Quantitative Cerebrovascular Analysis for Improved Prediction of Post-Stroke Complications
Source: Transl Stroke Res. 2026 Jul 7;17(4):76. doi: 10.1007/s12975-026-01465-2 (PMC13342120; doi:10.1007/s12975-026-01465-2)
Supplement: Supplementary file 1 — Supplementary Material 1 [file 12975_2026_1465_MOESM1_ESM.docx]

**Supplemental Material**

**Methods:**

Outcome definitions:

Early Neurological Improvement (ENI):

ENI is a well-established early marker of favorable long-term recovery, assessed using the NIHSS within 24–48 hours post-reperfusion ^33^. ENI is typically assessed within 24–48 hours post-treatment using the National Institutes of Health Stroke Scale (NIHSS). Although multiple definitions exist, ENI is most commonly defined as a reduction of ≥4 NIHSS points or an absolute NIHSS of 0–1. This definition is used in the present study and reflects a validated predictor of downstream functional outcomes.

**Supplemental Results:**

**
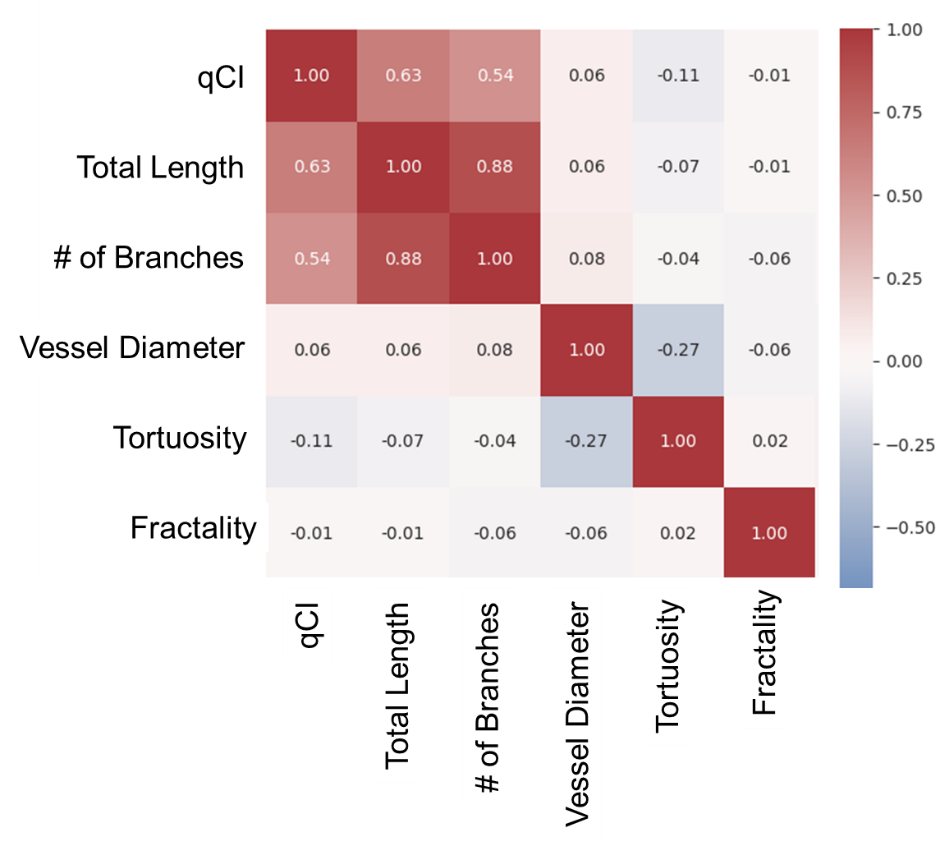
**

**Supplemental Figure S1. The correlation matrix for the quantitative morphology metrics, showing their intra-feature relationship and inter-dependency.**

## **Long-term Functional Outcomes (dichotomized 90-day mRS)**

For long-term functional outcome, dichotomized 90-day modified Rankin Scale (mRS good vs. poor), the morphology-enhanced model demonstrated superior discrimination and calibration compared with the baseline clinical model. Specifically, the morphology-informed model achieved an AUROC of approximately 0.69 compared to 0.59 for the clinical-only model, with consistent performance observed across both out-of-fold validation and independent test cohorts.

ENI:

Similarly, the morphology-integrated (MI) model demonstrated improved discrimination and calibration for ENI prediction relative to the clinical-only model (AUROC approximately 0.69 vs. 0.59). Better collateralization, reflected by higher qCI values, greater total vessel length, and increased branching complexity, was strongly associated with early neurological improvement. These results suggest that patient-specific cerebrovascular architecture captures physiologic reserve relevant to early recovery trajectories following EVT.

**Supplemental Table S1.** Net Reclassification Improvement (NRI) and Integrated Discrimination Improvement (IDI) analyses were performed to quantify the incremental predictive value of adding CTA-derived vascular morphology and collateral metrics to clinical models across outcomes. Positive NRI values indicate improved risk stratification, particularly driven by more accurate up-classification of true event cases.

| **Outcome** | **NRI (Total)** | **NRI (Events)** | **NRI (Non-Events)** | **IDI** |
| --- | --- | --- | --- | --- |
| **90-day mRS** | 0.336 | 0.217 | 0.119 | 0.0928 |
| **ENI** | 0.1366 | 0.0556 | 0.0811 | 0.0257 |

Supplemental Risk Stratification Analysis:

Risk band analyses for ENI and 90-day mRS further showed monotonic increases in observed outcome rates across low-, moderate-, and high-risk strata in the morphology-informed model, supporting its utility for individualized patient-level risk assessment.


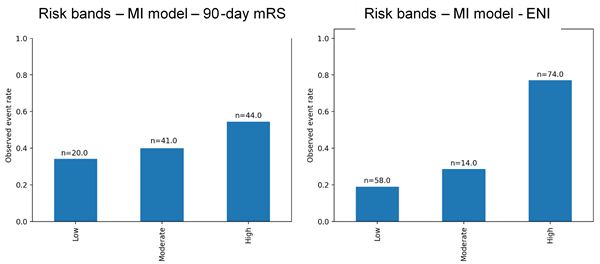


**Supplemental Figure S2. Risk band stratification for 90-day mRS and ENI (MI model).**
